# Transcriptomics using lung resection material to advance our understanding of COPD and idiopathic pulmonary fibrosis pathogenesis

**DOI:** 10.1183/23120541.00061-2024

**Published:** 2024-08-05

**Authors:** Kamini Rakkar, Dhruma Thakker, Michael A. Portelli, Ian Hall, Holger Schlüter, Ian Sayers

**Affiliations:** 1 Centre for Respiratory Research, NIHR Nottingham Biomedical Research Centre, School of Medicine, Biodiscovery Institute, University of Nottingham, Nottingham, UK; 2 Immunology and Respiratory Department, Boehringer Ingelheim Pharma GmbH & Co. KG, Biberach, Germany

## Abstract

COPD and idiopathic pulmonary fibrosis (IPF) are both chronic lung diseases with distinct pathologies. COPD is characterised by progressive airflow obstruction and encompasses emphysema leading to loss of alveolar structure and chronic bronchitis including inflammation [1]. IPF is a progressive fatal interstitial lung disease without effective treatment, characterised by fibrosis of the lung [2]. Risk factors such as smoking and symptoms such as shortness of breath are common to both. Although the clinical features are well defined and categorised, the differences and similarities between mechanistic pathways have not been fully investigated.

## Shareable abstract

**Genes involved in cell death, inflammation and viral infection are common to both COPD and IPF. A link to rheumatic disease is unique to COPD, and IPF-specific analyses showed increases in gene expression of keratins, collagens, mucins and MMPs.** https://bit.ly/3JoW73H

*To the Editor:*

COPD and idiopathic pulmonary fibrosis (IPF) are both chronic lung diseases with distinct pathologies. COPD is characterised by progressive airflow obstruction and encompasses emphysema leading to loss of alveolar structure and chronic bronchitis including inflammation [1]. IPF is a progressive fatal interstitial lung disease without effective treatment, characterised by fibrosis of the lung [2]. Risk factors such as smoking and symptoms such as shortness of breath are common to both. Although the clinical features are well defined and categorised, the differences and similarities between mechanistic pathways have not been fully investigated.

Recent studies have shown that both COPD and IPF show altered lung tissue gene expression compared to controls, with a subset of overlapping genes and disease mechanisms [3, 4].

In this study, we aimed to see whether lung tissue from COPD or IPF patients’ tissue resections could be used to identify gene and pathway expression differences between control and COPD or IPF lung tissue, and provide new mechanistic insight.

Lung tissue was obtained from the Royal Papworth Hospital research tissue bank. Informed consent was obtained and ethical approval was granted by the Royal Papworth Hospital Tissue Bank (Research Ethics Committee references 08/H0304/56+5 and 18/EE/0269). Tissue was taken from adjacent normal tissue from post-lobar resection or whole/partial-lung transplant procedures. Clinical and demographic information was obtained where possible. The tissue was rinsed in Tyrode's buffer and a piece for RNA extraction was placed in 1 mL RNAlater overnight at 4°C after which the RNAlater was removed, and the tissue frozen at −80°C. RNA was extracted using the Qiagen AllPrep DNA/RNA Mini Kit and sent to Oxford Genomics for RNA sequencing. All samples had RNA integrity numbers ≥8.

Gene expression was determined through stranded paired-end polyA RNA sequencing using Illumina version 1.5 chemistry and the NovaSeq6000 at a minimum of 25 million reads. Raw data were trimmed using Trimmomatic and aligned to the Genome Reference Consortium human build 37 assembly using STAR. All data processing and analysis was conducted as previously described [5]. Results were corrected for donor age, sex, smoking history and RNA extraction batch, and adjusted using the Benjamini–Hochberg procedure (false discovery rate 5%, p<0.05).

The Ingenuity Pathway Analysis (IPA) tool (Qiagen) was used to determine disease, pathway and upstream regulator effect associations for the differentially expressed genes as previously described [5].

Comparison of clinical characteristics between the control, COPD and IPF groups identified significant differences in male/female sex ratios, smoking history and lung function, as anticipated (figure 1a).

**FIGURE 1:**
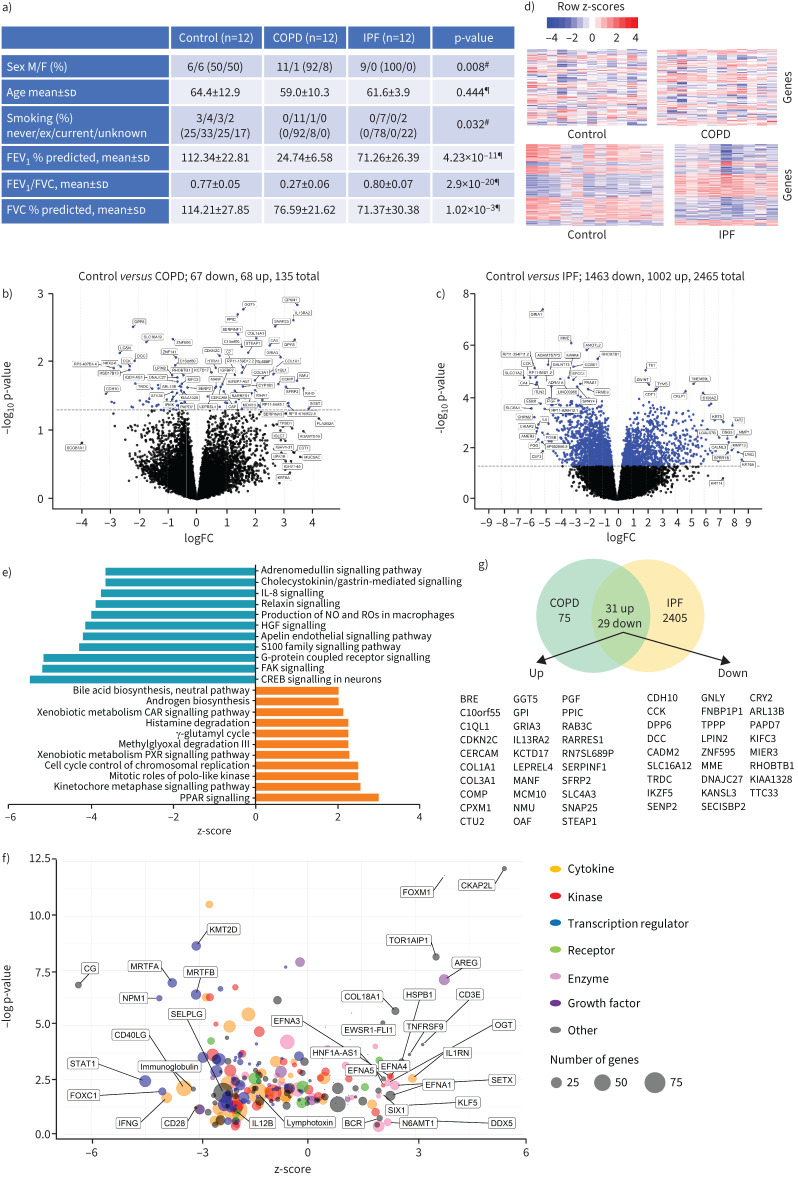
Donor information, differential gene expression and pathway analysis changes between control, COPD and idiopathic pulmonary fibrosis (IPF) groups. a) Tissue donor demographic information and analysis. Volcano plots and corresponding heatmaps for differential gene expression analysis between b) control and COPD and c) control and IPF groups with d) corresponding heatmaps. Results were corrected for donor age, sex, smoking history and RNA extraction batch, and adjusted using the Benjamini–Hochberg procedure (false discovery rate 5%; p<0.05). e) Ingenuity pathway analysis (IPA) for the control *versus* IPF differential gene expression dataset showing top activated (z-score >2, orange) and inhibited (z-score <2, blue) pathways. f) IPA for the control *versus* IPF differential gene expression dataset showing activated (z-score >2) and inhibited (z-score <2) upstream regulators (p<0.05). g) Overlapping genes between the differential expression analysis for the control *versus* COPD and control *versus* IPF datasets. FEV_1_: forced expiratory volume in 1 s; FVC: forced vital capacity; FC: fold change; ^#^: Chi-squared test; ^¶^: one-way ANOVA.

Gene expression analysis between the control and COPD groups identified a total of 135 differentially expressed genes. Genes such as interleukin receptor subunit α2 (*IL13RA2*), matrix proteins (*COMP, COL1A1* and *COL14A1*), growth regulators (*EGFL6, SOST* and *SFRP2*) and G-proteins (*RAB3C* and *GPR17*) showed logarithmically transformed fold changes (FC) all >2; whereas genes such as cadherin (*CDH10*) and those related to molecule processing (*GSTA3* and *HSD17B13*), phospholipid binding (*SYTL5*), potassium channels (*DPP6*) and cytoskeletal proteins (*LGSN*) all showed log FC < −2. (Figure 1b).

Gene expression analysis between the control and IPF groups resulted in a total of 2465 differentially expressed genes. The most notable overexpressed genes were related to keratins (*KRT5, KRT16, KRT15* and *KRT13*), collagen (*COL17A1*), mucin (*MUC5AC*) and matrix metalloproteinases (MMPs) (*MMP1, MMP13, MMP7* and *MMP8*), which all had log FC >5. Genes that were underexpressed were more diverse, such as protein/ion transport (*SLCO1A2* and *SLC6A4*), cytokines (*IL6, CSF3*), muscarinic receptor (*CHRM2*) and angiogenesis (*ESM1*), which all showed log FC < −5 (figure 1c).

IPA results from the control *versus* COPD gene expression dataset showed a small number of associations possibly due to the small number of differentially expressed genes and the individual subject heterogeneity observed in the heatmap (figure 1d). Only the wound healing signalling pathway and upstream regulators *SORL1* and *CG* were seen to be activated with a z-score >2 (data not shown). No pathways or regulators were found to be inhibited and there were no strongly linked diseases apart from those related to cancer.

IPA results from the control *versus* IPF dataset showed a greater number of associations. Pathways such as peroxisome proliferator-activated signalling, xenobiotic metabolism and cell cycle-related mechanisms were activated, and all had z-scores >2 (figure 1e, only top 10 pathways shown). There were a larger number of inhibited pathways such as those involved cytokine signalling (*IL8*, *IL17* and *IL6*), nitric oxide signalling, white blood cell signalling (natural killer cells and macrophages, which all had z-scores <2) (figure 1e, only top 10 pathways shown). Furthermore, there were also associations with disease, such as IPF (p=9.23×10^−18^), chronic pulmonary disease (p=3.71×10^07^), fibrosis (p=6.54×10^−19^) and cardiac hypertrophy (p=1.15×10^−4^). >60% of disease associations were cancer related (data not shown).

In the IPF dataset, upstream regulators or factors such as *COL18A1* and *IL1RN* were predicted to be active compared to factors such as *STAT1*, *IFNG* and *IL12B*, which were inhibited in the IPF tissue (figure 1f). Most inhibited factors tended to be transcription regulators or cytokines.

Investigation of the overlap between the COPD and IPF gene expression datasets showed that there were 60 common genes representing 44% of the total differentially expressed genes from the control *versus* COPD comparison and 2.4% of the total differentially expressed genes in the control *versus* IPF comparison (figure 1g). Of these, 31 genes were upregulated in both diseases such as *COL31A1, COL1A1* and *IL13RA2* and 29 genes were down regulated in both diseases such as *MME, DNAJC27* and *KIFC3*. There were no genes which were common to both datasets but showed gene expression in opposite directions.

IPA on overlapping genes showed processes such as “apoptosis” (p=0.002), “extracellular matrix organisation” (p=3.89 x10^−4^) and “necrosis” (p=0.001) were associated with the common upregulated genes, and processes such as “infection by RNA virus” (p=0.024) and “cell junction organisation” (p=0.005) were associated with the common downregulated genes. “Chronic inflammatory disorder” (p=0.015) was associated with both down- and upregulated genes, suggesting these may be common pathways/mechanisms to both diseases.

Interestingly, pathways involved in rheumatic disease (p=0.015) appeared to be associated only with genes differentially upregulated in the control *versus* COPD dataset (*CYP7B1, DPYS, HTRA1, IGFBP7, IGHD, SNAI2* and *TMEM266*), indicating genes in this pathway may be unique to COPD lung tissue and/or that rheumatic disease and COPD are strongly linked comorbidities. Over 100 common upstream regulators were identified, of which >70% had either *COL1A1* or *COL3A1* as their target molecule, indicating collagen is a key factor in both diseases, supporting evidence from previous studies [6, 7]. There were no upstream regulators identified by IPA that were unique to the COPD or IPF gene dataset.

This study aimed to further our mechanistic understanding of COPD and IPF *via* transcriptomics using lung resection tissue. Importantly, we show that there is a subset of genes that are differentially expressed in the same direction in both IPF and COPD, suggesting common causal pathways with associated opportunities for therapeutic intervention. In the COPD-specific analyses, we could not provide significant insight due to the small number of differentially expressed genes and inconclusive IPA, although a general increase in matrix-associated protein and growth regulator gene expression was seen and there was a strong link to rheumatic disease. This link has been extensively reported [8] and the genes highlighted in our study may provide novel avenues of exploration. The IPF-specific analyses supported previous literature with regards to increases in expression levels of genes implicated in IPF pathogenesis (*e.g.* elevated levels of keratins, collagens, mucins and MMPs) and, importantly, identified other genes for additional study [9–12].

Although this study would benefit from a larger discovery and a replication cohort due to its modest sampling, and while donor heterogeneity, especially in the COPD tissue, and incomplete comorbidity and medication data limit our ability to fully define common/different pathways between COPD and IPF, this study highlights some potential mechanistic pathways that are involved in both COPD and IPF, such as apoptosis, necrosis, chronic inflammation and viral infection. Similarly, the IPF analyses identified genes involved in transcriptional regulation and inflammation, supporting previously associated factors/pathways, such as MMPs and fibrosis. This study also provides confirmation of pathways and genes unique to COPD, which are involved in other diseases such as rheumatic disease. This study provides a foundation for further in-depth study of distinct and overlapping pathways contribution to these difficult to treat diseases.
